# Role of Histamine in Modulating the Immune Response and Inflammation

**DOI:** 10.1155/2018/9524075

**Published:** 2018-08-27

**Authors:** Anna Cláudia Calvielli Castelo Branco, Fábio Seiti Yamada Yoshikawa, Anna Julia Pietrobon, Maria Notomi Sato

**Affiliations:** Laboratory of Dermatology and Immunodeficiencies, LIM-56, Tropical Medical Institute, Department of Dermatology, School of Medicine, University of São Paulo, Sao Paulo, SP, Brazil

## Abstract

Inflammatory mediators, including cytokines, histamine, bradykinin, prostaglandins, and leukotrienes, impact the immune system, usually as proinflammatory factors. Other mediators act as regulatory components to establish homeostasis after injury or prevent the inflammatory process. Histamine, a biogenic vasoactive amine, causes symptoms such as allergies and has a pleiotropic effect that is dependent on its interaction with its four histamine receptors. In this review, we discuss the dualistic effects of histamine: how histamine affects inflammation of the immune system through the activation of intracellular pathways that induce the production of inflammatory mediators and cytokines in different immune cells and how histamine exerts regulatory functions in innate and adaptive immune responses. We also evaluate the interactions between these effects.

## 1. Introduction

Histamine and its receptors represent a complex system of immunoregulation with distinct effects mediated by four GPCRs (G protein-coupled receptors HRs 1–4) and their differential expression, which changes according to the stage of cell differentiation and microenvironmental influences. Several host factors, in addition to genetic factors, may influence histamine/receptor effects, including the microbiota, gender, ageing, autoimmune diseases, inflammatory skin, cancer, gut, and pulmonary diseases.

Inflammatory conditions (e.g., allergy, asthma, and autoimmune diseases) have long been thought to be mainly mediated by the activation of histamine receptor 1 (H1R). However, in the treatment of diseases such as chronic pruritus, asthma, and allergic rhinitis, the use of selective H4R ligands and/or modulation of H1 and H4 receptor synergism may be more effective for such pathophysiological conditions. Recent evidence strongly suggests that H4R ligands might be exploited as potential therapeutics in allergy, inflammation, autoimmune disorders, and possibly cancer. Overall, exploiting the impact of histamine on innate and adaptive immune responses may be helpful for understanding receptor signaling and trends during inflammation or regulation.

Histamine shows a dichotomous nature, whereby it is able to promote inflammatory and regulatory responses that contribute to pathological processes, such as allergy induction, as well as homeostatic functions, such as intestinal regulation. In this review, we summarize recent findings about the regulation of the immune response by histamine. A general overview of the immune cascades triggered by histamine receptor activation is provided.

## 2. Histamine and Histamine Receptors

Histamine (2-[3H-imidazol-4-yl]ethanamine) is an important chemical mediator that causes vasodilation and increased vascular permeability and may even contribute to anaphylactic reactions [1]. It also acts on several physiological functions, such as cell differentiation, proliferation, haematopoiesis, and cell regeneration. Synthesis of histamine occurs through decarboxylation of the amino acid histidine by the enzyme L-histidine decarboxylase (HDC), which is expressed in neurons, parietal cells, gastric mucosal cells, mast cells, and basophils; degradation of histamine is mediated by the enzyme diamine oxidase (DAO) and histamine N-methyltransferase (HNMT), which catalyses histamine deamination [2, 3]. HNMT is expressed in the central nervous system, where it may play a critical regulatory role because its deficiency is related to aggressive behaviour and abnormal sleep-wake cycles in mice [4].

The pleiotropic effects of histamine are mediated by 4 histamine receptors (HRs), H1R, H2R, H3R, and H4R, which are G protein-coupled receptors. The active and inactive conformations of these receptors coexist in equilibrium. Agonists of these receptors stabilize the active conformation, whereas antagonists stabilize the inactive conformation. Curiously, the ageing process impairs expression or activity of HRs, and the enzymes HDC and DAO may contribute to the progression of allergic reactions and various neurodegenerative disorders [5]. Chronic itch in the elderly is a common problem that is often multifactorial due to physiological changes in ageing skin, including impaired skin barrier function, and changes in immunological, neurological, and psychological systems associated with age.

H1R is expressed in various cell types, such as neurons, endothelial cells, adrenal medulla, muscle cells, hepatocytes, chondrocytes, monocytes, neutrophils, eosinophils, DCs, T cells, and B cells. H1R signaling results in the following: synthesis of prostacyclins; activation of platelet factor; synthesis of nitric oxide, arachidonic acid and its metabolites, and thromboxane; and contraction of smooth muscle cells. In addition, H1R activation leads to increased chemotaxis of eosinophils and neutrophils at the site of inflammation, higher functional capacity of antigen-presenting cells (APCs), activation of Th1 lymphocytes, and decreased humoral immunity but the promotion of IgE production [6]. As expected for such biological actions, H1R antagonists, including pyrilamine, fexofenadine, diphenhydramine, and promethazine, are commonly used for the treatment of allergic symptoms.

Signaling via H1R leads to the activation of intracellular transcription factors, such as IP3 (inositol triphosphate), PLC (phospholipase C), PKC (protein kinase C), DAG (diacylglycerol), and Ca^2+^. Recently, H1R and H4R signaling was implicated in MAPK signaling and cAMP accumulation, leading to increased proinflammatory gene expression [7]. In addition, activation of H1R is important for the generation of Th1 responses, whereas H2R regulates Th2 responses. Mice genetically deficient for H1R (H1R−/−) have an exacerbated Th2 profile due to a decrease in Th1 responses [8]. In addition, H1R was demonstrated in an experimental allergy model to play a critical role together with histamine in orchestrating recruitment of Th2 cells to the site of allergic lung inflammation [9].

H2R is expressed by parietal cells of the gastric mucosa, muscle, epithelial, endothelial, neuronal, hepatocyte, and immune cells. H2R antagonizes some of the effects mediated by H1R and leads to the relaxation of smooth muscle cells, causing vasodilation. H2R activation regulates several of the functions mediated by histamine, including cardiac contraction, gastric acid secretion, cell proliferation, and differentiation [10]. It also acts as a suppressor molecule in DCs, increasing IL-10 production [11]. One recent study demonstrated that histamine acts on H2R and induces inhibition of leukotriene synthesis in human neutrophils through cAMP-dependent protein kinase (PKA) signaling [12]. In a murine lung inflammation model, H2R loss has an effect on invariant natural killer T (iNKT) cells, aggravating local inflammation [13].

In monocyte-derived DCs from healthy adult subjects, H2R activation counterbalances the Toll-like receptor (TLR) response, leading to inhibition of CXCL10, IL-12, and TNF-*α* stimulation of IL-10, which is likely associated with Th2 polarization [14]. Mechanistically, inhibition of TLR-associated NF-*κ*B and AP-1 pathways occurs due to cAMP activation downstream of H2R activation [14].

While the activation H1R and H2R mainly accounts for mast cell- and basophil-mediated allergic disorders [15], H3R functions were identified in the central nervous system and peripheral and presynaptic receptors to control the release of histamine and other neurotransmitters. The asymmetry of histamine via H3R inhibits the acetylcholine released in the mouse cortex, which controls neurogenic inflammation by inhibiting cAMP formation and Ca^2+^ accumulation [16]. H3R knockout mice exhibit an obese phenotype, suggesting that H3R regulates insulin resistance and leptin release, as well as a decrease in homeostatic energy, the cellular process for coordinating homeostatic regulation of food intake (energy inflow) and energy expenditure (energy outflow), as associated with the UCP1 and UCP3 genes [17]. H3R expression may be associated with bronchoconstriction, pruritus (without involvement of mast cells), increased proinflammatory activity, and antigen-presentation capacity [18].

Neuromodulation and the waking state are related to histaminergic neurons. The waking state is maintained by continual activation of aminergics (such as histamine, dopamine, noradrenaline, and acetylcholine). Three subtypes of HRs are widely distributed in the brain, not only on neurons but also on astrocytes and blood vessels. Positive-allosteric modulators of GABA_A_ receptors acting on histamine neurons in the posterior hypothalamus induce a natural NREM-like sleep [19]. Targeting the histamine and noradrenergic systems may aid in the design of more precise sedatives and, at the same time, may reveal more about the natural sleep-wake circuitry [19]. In fact, there is a potential utility of histamine H3R antagonist/inverse agonists for CNS disorders. An experimental study showed that when subjected to lipopolysaccharide (LPS) challenge, histamine inhibits the injurious effect of microglia-mediated inflammation by protecting dopaminergic neurons, highlighting the down-modulatory ability of histamine and/or HR agonists. This finding may be useful for the development of new therapeutic approaches to treat neurodegenerative disorders [20].

H4R is preferentially expressed in the intestine, spleen, thymus, bone marrow, peripheral haematopoietic cells, and cells of the innate and adaptive immune systems. Expression of H4R is regulated by stimulation with IFN, TNF-*α*, IL-6, IL-10, and IL-13, leading to inhibition of cAMP accumulation and activation of MAPK (mitogen-activated protein kinases) by H4R. Activation of this receptor causes chemotaxis in mast cells and eosinophils, leading to an accumulation of inflammatory cells and control of cytokine secretion by DC and T cells. H4R is also involved in increased secretion of IL-31 by Th2 cells [21]. Treatment of mice with the H4R antagonist JNJ7777120 attenuates pruritus in response to histamine, IgE, and compound 48/80, and its inhibitory effect is greater than that observed with H1R antagonists [22]. The use of this synthetic H4R antagonist in a murine encephalomyelitis model resulted in an increase in the clinical and pathological signs of the disease, suggesting a modulatory role [23].

HRs are present on tumour cells, making them sensitive to variations in histamine. High levels of histamine are associated with bivalent behaviour in the regulation of several tumours (i.e., cervical, ovarian, vaginal, uterine, vulvar, colorectal, and melanoma cancers) by promoting or inhibiting their growth [24]. The presence of H3R and H4R in human mammary tissue suggests that H3R may be involved in regulating breast cancer growth and progression [25], emphasizing the possible use of antihistamines as adjuvants in cancer chemotherapy.

In HDC-deficient mice, a decrease in H4R expression on iNKT cells is associated with lower production of IL-4 and IFN-*γ* by those cells, which demonstrates regulation between these factors [26]. Several studies have shown that histamine is involved in regulating the function of DCs [27], such as by potentiating antigen endocytosis, inducing intracellular Ca^2+^ mobilization, promoting F-actin polymerization in immature DCs derived from monocytes [28], and promoting expression of MHC class II molecules. Strikingly, cross-presentation, the ability to drive MHC II-associated antigens towards the MHC I pathway, is blocked by H3R/H4R antagonists. Histamine also acts on T cell polarization by inhibiting IFN-*γ* or LPS-driven IL-12 production in a H1R/H2R dependent manner [29]. H4R has a modulatory role in APCs (DCs and monocytes) by exerting anti-inflammatory action and reducing IL-12 and CCL2 production [28, 30].

Asthma is prevalent in males during childhood but is more frequent in females during adolescence and adulthood. Furthermore, allergic diseases are common in women of childbearing age. Both asthma and atopic conditions may worsen, improve, or remain the same during pregnancy. Female hormones, such as estrogen, can modulate the inflammatory response, and histamine receptors can differ between males and females, which might explain the different incidence of allergy between the sexes [31]. For example, H2R and H3R are highly expressed in female compared to male rats and are downregulated in ovariectomized females, whereas H1R is equally expressed in both sexes [32].

**Figure 1:**
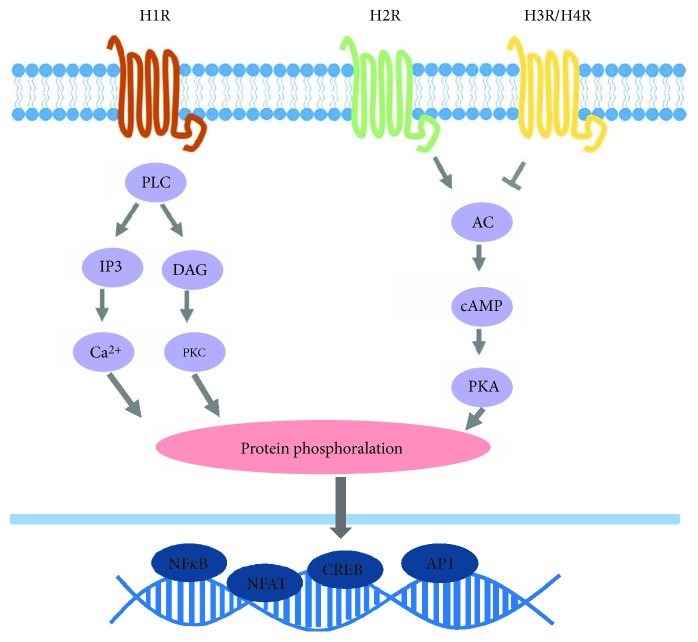
Intracellular activation cascades triggered by histamine receptors (HRs). The pleiotropic effects of histamine are mediated by four histamine receptors: H1R, H2R, H3R, and H4R, which are G protein-coupled receptors. Signaling via H1R leads to activation of intracellular transcription factors, such as PLC (phospholipase C), IP3 (inositol triphosphate), PKC (protein kinase C), DAG (diacylglycerol), and Ca^2+^. H2R signaling acts through activation of adenylyl cyclase (AC), which increases cyclic adenosine monophosphate (cAMP) levels and activates protein kinase A (PKA), while H3R and H4R inhibit this cascade. Those intracellular signaling pathways culminate into protein phosphorylation and transcription of nuclear factor such as nuclear factor kappa B (NF-*κ*B), nuclear factor of activated T-cells (NFAT), cAMP response element binding protein (CREB), and activator protein 1 (AP-1).

The cascades and effects of different histamine receptors are summarized in Figure 1 and Table 1.

## 3. Histamine Stimulates Inflammation

Inflammatory mediators are molecules produced by activated cells that intensify and prolong the inflammatory response. Histamine is a potent inflammatory mediator, commonly associated with allergic reactions, promoting vascular and tissue changes and possessing high chemoattractant activity. The binding of histamine to eosinophil H4R induces increased expression of macrophage-1 antigen (Mac1) and ICAM-1 adhesion molecules, in addition to promoting actin filament rearrangement [33, 34]. These events favour the migration of eosinophils from the bloodstream to the site of inflammation. In mast cells, the binding of histamine to this same receptor promotes the intracellular release of calcium and recruitment of mast cells into tissues [35]. Mast cells from H4R-knockout mice lose the ability to migrate against a histamine gradient [35]. Recruitment of these cells to sites of inflammation amplifies the inflammatory reactions mediated by histamine and may favour the establishment of a chronic inflammatory response. In experimental models, histamine drives colitis via HR4 by promoting granulocyte infiltration into the colonic mucosa [36].

Histamine also modulates the inflammatory response by acting on other cellular populations, in human lung macrophages, binding of histamine to H1R induces production of the proinflammatory cytokine IL-6 and *β*-glucuronidase [37, 38], a marker of exocytosis, and the release of lysosomal enzymes is associated with epithelial damage and rupture of the basement membrane [39]. Together, these events suggest that histamine may contribute to the maintenance of inflammatory conditions in the airways. In contrast, activation of H2R in rat peritoneal macrophages inhibits production of TNF-*α* and IL-12 when stimulated with LPS [40].

In an animal model of allergic airway inflammation, H4R-knockout mice present lower inflammation, reduced pulmonary infiltrate of eosinophils and lymphocytes and an attenuated Th2 response [41]. The contribution of histamine to the induction of airway inflammation is also due to its effect on nonimmune cells. In nasal fibroblasts, there is a dose-dependent increase in IL-6 production in response to histamine stimulation [42]. This inflammatory mediator increases expression of phosphorylated p38, pERK, and pJNK and induces NF-*κ*B activation. Treatment with H1R antagonists reduces expression of phosphorylated p38 and NF-*κ*B and, consequently, IL-6 production.

Blocking H4R in a model of pulmonary fibrosis alleviates the inflammatory response, reducing COX2 expression and activity, leukocyte infiltration, production of TGF-*β* (profibrotic cytokine), and collagen deposition [43]. Histamine also activates pulmonary epithelial cells. Histamine binding to H1R enhances TLR3 expression in these cells, and treatment with the TLR agonist poly(I:C), along with histamine, potentiates NF-*κ*B phosphorylation and IL-8 secretion, indicating an increased response of epithelial cells to microbial ligands [44].

In the nervous system, microglial activation is regulated by histamine in a dose-dependent manner, which leads to the production of proinflammatory cytokines, such as IL-6 and TNF-*α* [45]. This activation is mediated via H1R, and H4R and is dependent on MAPK and PI3K/AKT cascades. In addition to inducing iNOS expression and NO production, histamine promotes the loss of mitochondrial membrane potential and the production of ROS in microglia by binding to these same receptors [46, 47]. Overall, the accumulation of these cytokines and proinflammatory molecules can be deleterious, leading to nerve damage.

Histamine also modulates the response of DCs. Stimulation of immature DCs induces expression of CD86, CD80, and MHC class II, increasing the efficiency of T cell activation [11]. In the presence of histamine, DCs exhibit a higher production of IL-6, IL-8, and CCL2 as well as induced expression of IL-1*β*, CCL5, and CCL4. However, the H2R pathway promotes IL-10 production and inhibits IL-12 synthesis by immature DCs, favouring the development of a Th2 response profile [29]. This modulation of cytokine production, suggests that histamine indirectly alters the Th1/Th2 balance through the stimulation of DCs. In a food allergy model, simultaneous blockage of H1R and H4R inhibited the development of intestinal inflammation and diarrhoea when the animals were exposed to the allergen by suppressing histamine-mediated DC antigen presentation and chemotaxis [48].

The Th1/Th2 balance is directly regulated by histamine, as described above. Th1 cells display higher H1R expression, and their binding to histamine promotes activation of Th1 responses, potentiating IFN-*γ* production [49]. In contrast, Th1 and Th2 responses are inhibited by histamine stimulation via H2R. Histamine also alters the response of other subpopulations of lymphocytes. Activation of CD8+ T cells via H4R induces secretion of IL-16, a chemoattractant molecule for CD4+ cells such as monocytes and DCs [50]. In addition, histamine stimuli induces IL-17 production in human Th17 cells, suggesting the contribution of this inflammatory mediator to the activation of lymphocytes present in skin lesions in atopic dermatitis and psoriasis [51].

Other evidence, indicates that histamine plays an important role in inflammatory skin diseases. When stimulated via H4R, the NK cells present in skin lesions, increase expression of the chemokines CCL3 and CCL4, favouring cell recruitment to injured tissue [52]. As H4R knockout mice display a lower influx of inflammatory cells and less cell proliferation at the lesion sites, H4R is associated with the inflammatory response in atopic dermatitis [53]. H4R is also involved in NK cell recruitment and induction of CCL17 production by DCs at lesion sites in murine models of atopic dermatitis, contributing to increased local inflammation [54]. In an experimental model of pruriginous dermatitis, H4R blockage decreases itching because the activation of this receptor is involved in increased IL-31 secretion by Th2 cells [21, 55]. Moreover, blocking H4R improves skin lesions and reduces the number of mast cells at lesion sites [55].

Within the context of vascular inflammatory diseases, histamine produced by the tunica intima stimulates the monocytes present in atherosclerotic plaques to express CCL2 and its receptor CCR2, via activation of H2R [56]. Furthermore, higher production of IL-6 and adhesion molecules, such as ICAM-1 and VCAM-1, occurs in endothelial cells stimulated by histamine, thereby favouring progression of the disease [56, 57]. In an experimental model, the absence of H1R reduced the development of atherosclerosis, whereas the absence of H2R exerted the opposite effect [58].

## 4. Regulatory and Immunomodulatory Functions of Histamine

As discussed above, the pleiotropic effects of histamine are a consequence of the existence of four different receptors that belong to the same family of G-coupled proteins and trigger different signaling cascades; these receptors are differentially distributed across tissues and cells [59]. Therefore, in addition to its classical roles in the inflammatory process, histamine is recognized as a key player in immune regulation.

Although histamine is commonly associated with skin inflammation processes, for example, allergic dermatitis [60], it may play a regulatory role in other clinical conditions such as psoriasis, which is a multifactorial Th1/Th17-driven inflammation of the skin [61]. In a murine psoriasis-like model induced by imiquimod administration, Kim et al. showed that the H4R agonist 4-methylhistamine ameliorated the clinical scores of psoriatic mice due to repression of Th1 cytokines and simultaneous induction of Treg cells [62]. These findings suggest that histamine targeting has pharmacological potential. In addition, histamine participates in the wound-healing process by increasing the viability of rat wound fibroblasts and promoting the production of TGF-*β* in an H1R-dependent manner [63].

The contribution of histamine to inflammatory neurological diseases, such as multiple sclerosis, is controversial: although H1R and H2R appear to favour the inflammatory response in brain lesions [64], H3R dampens neuroinflammation, mainly by modulating the production of chemokines and maintaining the integrity of the blood-brain barrier [65] in a murine model of multiple sclerosis, experimental autoimmune encephalomyelitis (EAE). Pharmacological blockage of H4R exacerbates EAE, as observed by increased recruitment of inflammatory mediators, larger lesion areas, and enhanced activation of T cells, indicating that H4R also plays a regulatory role in a neurological context [23]. In a murine model of the motor neuron disorder amyotrophic lateral sclerosis (ALS), microglia from SOD1-G93A mice, which spontaneously develop an ALS phenotype, appear to be responsive to histamine through H1R and H4R, leading to a reduction in the inflammatory state. The findings suggest deregulation of the histaminergic pathway in ALS patients [66].

Regardless, the role of histamine in gut homeostasis maintenance is clearly essential, as exemplified in a murine model of HDC deficiency. Yang et al. showed that HDC^−/−^ mice were prone to develop inflammation and had a higher tumour burden at mucosal sites (intestine and skin) in models of chemically induced carcinogenesis but that their phenotype was attenuated by histamine administration [67]. Most HDC expression in these tissues was found in immature myeloid cells (IMCs), rather than in resident mast cells; curiously, histamine administration, acting through H1R and H2R, was required to promote terminal differentiation of IMCs into monocytes and neutrophils [67].

Expression levels of HDC and H2R are positively associated with increased survival in colorectal cancer patients [68], and some epidemiological studies support the association of atopy with the reduced incidence of some cancers [69, 70], reinforcing a possible role of histamine in blocking tumour development.

In addition to tumour biology, histamine has a regulatory function in gut inflammatory diseases, such as in a murine trinitrobenzene sulfonic acid- (TNBS-) induced model of colonic inflammation, which promotes a delayed hypersensitivity reaction that resembles the phenotype [71]. By employing H4R-deficient mice, Wunschel et al. demonstrated that histamine protects animals from an exacerbated inflammatory reaction in the colon, as illustrated by enhanced production of chemokines/cytokines and loss of intestinal architecture, which ultimately leads to death [72]. Strikingly, Schirmer et al. reported the opposite effect using a similar mouse background (H4R^−/−^ mice), whereby histamine worsened dextran sodium sulfate- (DSS-) induced colitis and pharmacological inhibition of H4R ameliorated the health of the mice [73]. Differences between the models, such as the chemical stimulation used and the route of administration, may account for the opposite results. Overall, the response that prevails in humans requires further investigation.

Recently, the microbiota was revealed to be very important for gut pathophysiology. Indeed, the microbial community in the intestine shapes the health status of the host and its susceptibility to a broad range of local and systemic diseases [74]. The microbiota supplies the host with a number of metabolites, such as short-chain fatty acids, tryptophan metabolites and histamine [75, 76], and a recent work highlights the key role of microbe-derived histamine in the host response.

For example, histamine-secreting bacteria (*Escherichia coli*, *Lactobacillus vaginalis*, and *Morganella morganii*) are found at higher frequency in faecal samples from asthmatic patients, with a possible contribution to their atopic phenotype [76, 77], as microbial-derived histamine is indistinguishable from the human-produced form.

*Lactobacillus reuteri* is a member of the gut microbiota that belongs to the Firmicutes family; HDC gene expression by this commensal bacterium confers the ability to produce histamine from histidine [78]. Microbial histamine modulates the inflammatory response of the human monocyte cell line THP-1, mainly by acting through H2R, to promote activation of the cAMP/PKA cascade and block of ERK signaling [79]. *L. reuteri*-derived histamine relies on H2R to promote its regulatory function in the intestine [80]. Interestingly, histamine modulates the response of myeloid and plasmacytoid DCs to LPS, favouring IL-10 production over the secretion of inflammatory cytokines [14] and enhancing their ability to phagocytose soluble antigens and upregulate expression of the costimulatory molecules CD86 and inducible costimulator ligand (ICOS-L) [81]. Although the effects depend on H2R and cAMP, histamine requires the participation of exchange proteins activated by cAMP (Epac) in DCs, as opposed to PKA in monocytes, indicating that these signaling cascades are cell type specific [14].

In addition to H2R agonism, *L. reuteri* simultaneously blocks H1R signaling by secreting the enzyme diacylglycerol kinase (DGK). H1R leads to the production of inflammatory mediators by triggering PKC-dependent NF-*κ*B activation [82]. PKC activation, in turn, requires the cofactor diacylglycerol (DAG); DGK degrades DAG into phosphatidic acid. Thus, *L. reuteri*-derived DGK interrupts H1R signaling and blocks the inflammatory effects of histamine but preserves H2R-associated immunomodulation [83].

*L. reuteri* administration protects HDC-deficient mice from colon tumours in a model of intestinal carcinogenesis with azoxymethane/dextran sodium sulfate (AOS/DSS) administration. *L. reuteri* produces or restores the histamine pool in the intestine, lowering recruitment of immature myeloid cells and production of inflammatory cytokines, which blocks tumour development [68]. Importantly, *L. reuteri* only produces histamine; therefore, it exerts regulatory activity regardless of whether the precursor (histidine) is exogenously provided, for example, by food intake [80]. This highlights the critical interplay between the host immune response, the microbiota, and environmental (dietary) influences on biological outcomes.

In addition to *L. reuteri*, other bacteria have probiotic potential. Another lactobacilli, *Lactobacillus rhamnosus*, is a source of histamine that promotes a regulatory Foxp3-T cell response profile in intestinal Peyer patches while dampening Th1 polarization in an H2R-dependent fashion [14].

The lung is a classical mucosal site under histamine control. Although histamine is associated with deleterious inflammation in asthmatic patients, triggering airway hyperresponsiveness and remodelling [84, 85], recent evidence suggests that the contribution of histamine to pulmonary homeostasis is not straightforward and depends on the receptor and cell type involved. H1R antagonism is well known for its beneficial effects in asthma management [86]; however, H2R deficiency or pharmacological blockade worsens lung inflammation in OVA-sensitized mice due to increased activation of iNKT cells, which promotes recruitment of macrophages and neutrophils and production of IL-4, IL-17, and IFN-*γ* by T cells [13].

Although the regulatory properties of histamine reveal a promising therapeutic potential, histamine may also exert deleterious effects in some clinical conditions. For example, histamine immunomodulation might contribute to higher susceptibility to sepsis in diabetic mice. Mast cell-derived histamine is increased in diabetic settings and reduces neutrophil recruitment due to repression of CXCR2 [87]. In addition, histamine may impair the oxidative burst of neutrophils [88]. Because neutrophils produce histamine once stimulated [89], a negative feedback loop is established, attenuating the microbicidal mechanism against invading bacteria.

## 5. Conclusion

**Figure 2:**
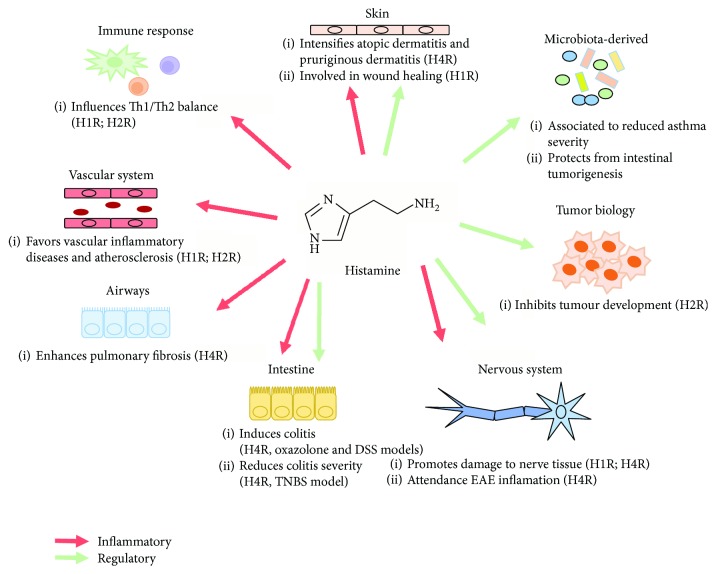
Inflammatory and regulatory functions of histamine on different body sites. Histamine plays dual functions according to the cell type and the receptor. As an inducer of inflammation, histamine can contribute to pulmonary fibrosis, cardiovascular diseases and atherosclerosis, atopic dermatitis, central nervous system damage, and colitis in some experimental models, besides favoring the polarization of the immune response to a Th1 profile. On the other hand, histamine can regulate inflammation in models of experimental autoimmune encephalomyelitis (EAE) and colitis, favor wound healing in skin lesions, and inhibit tumour development. Also, microbiota-derived histamine can regulate the inflammatory picture of asthma. Red arrows indicate proinflammatory action; green arrows indicate regulatory action of histamine.

Histamine research is an attractive perspective for the potential therapeutic exploitation of new drug targets. The main actions of histamine in controlling the immune response are summarized in Figure 2. The pleiotropic actions of histamine due to the different natures of its receptors allow this simple molecule to exert broad and oppose effects on the immune system, highlighting the importance of a fine-tune control that promotes a homeostatic environment in the body, balancing important inflammatory reactions to host protection as well as immunomodulation. The findings about the role of histamine in carcinogenesis, allergic regulation, and even behavioural regulation underscore histamine as a remarkable therapeutic target. However, as HRs are widely expressed in the body and change during cell differentiation and ageing and even differ between sexes, complex histamine/receptor agonism/antagonism should be exploited for therapeutic approaches with caution. New insight about the role of histamine in different body sites and cell types may result in more targeted therapies. Overall, recent findings about the microbial contribution to histamine homeostasis add a new layer of complexity to the picture. Ideal histaminergic therapy should be composed of a mixture of agonists and antagonists that would avoid deleterious side effects. Future studies may improve our understanding of the histamine network in organisms and its paradoxical nature.

## Figures and Tables

**Table 1:** Immunological functions mediated by histamine receptors signaling.

Receptor	Expression	Intracellular signaling	Immunological activity
H1R	Endothelial cells, nerve cells, epithelial cells, neutrophils, eosinophils, monocytes, macrophages, DCs, and T and B cells	PLC, PIP2, DAG, IP3, Ca^2+^, and PKC	Allergic reactions and inflammation, histamine release, eosinophil and neutrophil chemotaxis, antigen presentation ability, Th1/IFN-*γ* activity, and recruitment of Th2 cells; decreases humoral immunity and IgE production

H2R	Endothelial cells, nerve, epithelial, neutrophils, eosinophils, monocytes, macrophages, DCs, and T cells and B	Adenyl cyclase, cAMP, PKA, CREB, and EPAC	Increases IL-10 production and humoral immunity; decreases cellular immunity; inhibits Th2 cells and cytokines, chemotaxis of eosinophils, and neutrophils; suppresses IL-12p70 of MoDCs

H3R	Histaminergic neurons, monocytes, eosinophils	Inhibitor of adenyl cyclase and cAMP; increases levels of Ca^2+^	Control of neurogenic inflammation, increased proinflammatory activity, and antigen presentation capacity

H4R	Eosinophils, DCs, Langerhans cells, neutrophils, T cells, basophils, and mast cells	Inhibitor of adenyl cyclase and cAMP; increases levels of Ca^2+^	Affects pDC and mDC functions, Th1/Th2 differentiation, eosinophil and mast cell chemotaxis, IL-6 production, leukotriene B4, and migration of T*γ*/*δ* cells; increases IL-17 secretion by Th17 cells, and regulatory T recruitment; suppresses IL-12p70 of MoDCs
